# Deep learning analysis of exercise stress electrocardiography for identification of significant coronary artery disease

**DOI:** 10.3389/frai.2025.1496109

**Published:** 2025-03-17

**Authors:** Hsin-Yueh Liang, Kai-Cheng Hsu, Shang-Yu Chien, Chen-Yu Yeh, Ting-Hsuan Sun, Meng-Hsuan Liu, Kee Koon Ng

**Affiliations:** ^1^Division of Cardiology, Department of Medicine, China Medical University Hospital, Taichung, Taiwan; ^2^Department of Biomedical Imaging and Radiological Science, China Medical University, Taichung, Taiwan; ^3^Artificial Intelligence Center, China Medical University Hospital, Taichung, Taiwan; ^4^School of Medicine, China Medical University, Taichung, Taiwan; ^5^Department of Neurology, China Medical University Hospital, Taichung, Taiwan

**Keywords:** exercise stress electrocardiography, coronary artery disease, deep learning, multimodal approach, feature variable, artificial intelligence, clinical screening, convolutional recurrent neural network

## Abstract

**Background:**

The diagnostic power of exercise stress electrocardiography (ExECG) remains limited. We aimed to construct an artificial intelligence (AI)-based method to enhance ExECG performance to identify patients with significant coronary artery disease (CAD).

**Methods:**

We retrospectively collected 818 patients who underwent both ExECG and coronary angiography (CAG) within 6 months. The mean age was 57.0 ± 10.1 years, and 614 (75%) were male patients. Significant coronary artery disease was seen in 369 (43.8%) CAG reports. We also included 197 individuals with normal ExECG and low risk of CAD. A convolutional recurrent neural network algorithm, integrating electrocardiographic (ECG) signals and features from ExECG reports, was developed to predict the risk of significant CAD. We also investigated the optimal number of inputted ECG signal slices and features and the weighting of features for model performance.

**Results:**

Using the data of patients undergoing CAG for training and test sets, our algorithm had an area under the curve, sensitivity, and specificity of 0.74, 0.86, and 0.47, respectively, which increased to 0.83, 0.89, and 0.60, respectively, after enrolling 197 subjects with low risk of CAD. Three ECG signal slices and 12 features yielded optimal performance metrics. The principal predictive feature variables were sex, maximum heart rate, and ST/HR index. Our model generated results within one minute after completing ExECG.

**Conclusion:**

The multimodal AI algorithm, leveraging deep learning techniques, efficiently and accurately identifies patients with significant CAD using ExECG data, aiding clinical screening in both symptomatic and asymptomatic patients. Nevertheless, the specificity remains moderate (0.60), suggesting a potential for false positives and highlighting the need for further investigation.

## Introduction

Ischemic heart disease is the major cause of mortality worldwide. Recent findings from the global disease burden study indicate that ischemic heart disease caused more than 9 million deaths in 2021 (Vaduganathan et al., 2022; Malakar et al., 2019). Early diagnosis is crucial because lifestyle modification and medical intervention improve life quality and prolong survival (Knuuti et al., 2020).

The workup of a patient presented with suspected coronary artery disease (CAD) involves history taking, physical examination, and initial examinations. Possible CAD is further evaluated using many noninvasive test modalities, including exercise stress electrocardiography (ExECG), stress echocardiography, stress nuclear myocardial perfusion imaging, cardiovascular magnetic resonance imaging, and coronary computed tomography angiography (CCTA). Among them, ExECG, which has been used for >60 years, is a safe and affordable test for suspected CAD. However, although several ExECG scores, such as the Duke treadmill score, have been developed to improve diagnostic accuracy, the diagnostic power of ExECG remains limited with an area under the receiver operating characteristics curve (AUC) of 0.72–0.76 (Shaw et al., 1998).

Artificial intelligence (AI) has been applied in many disease models (Yadav and Jadhav, 2019; Movassagh et al., 2023; Alzubi et al., 2021; Kose et al., 2021). Given the limitations in the diagnostic accuracy of ExECG, AI offers the potential to overcome these challenges by detecting subtle patterns in ExECG data that might be missed by conventional interpretation methods. AI-enabled ExECG algorithms, which utilize various models and datasets to enhance accuracy, efficiency, and applicability of CAD prediction, have been published, with AUC, sensitivity, and specificity of 0.73–0.78, 0.25–0.85, and 0.43–0.97, respectively (Babaoglu et al., 2009; Babaoğlu et al., 2010; Yilmaz et al., 2023; Lee et al., 2022). A hybrid convolutional neural network (CNN)–long short-term memory (LSTM) architecture has been shown to effectively process and analyze electrocardiography (ECG) (Banerjee et al., 2020; Cheng et al., 2021). We hypothesized that the application of a hybrid CNN–LSTM model in ExECG might accurately and efficiently identify patients with significant CAD. To test this hypothesis, we conducted a retrospective study of ExECG in patients who underwent invasive coronary angiography (CAG) and those with normal ExECG to develop and validate a deep learning AI model to predict significant CAD.

The primary objectives of this study were to: (1) develop a hybrid CNN–LSTM algorithm that integrates ECG signals and ExECG features to improve the diagnostic accuracy of ExECG for significant CAD; (2) optimize the number and weighting of ECG signal segments and features to maximize model performance; and (3) evaluate the model’s efficiency in generating results. Our findings suggest that a multimodal AI algorithm leveraging deep learning can rapidly and accurately detect significant CAD from ExECG data, delivering results within one minute post-test. This advancement holds potential to enhance clinical screening for CAD in both symptomatic and asymptomatic patients.

### Materials and methods

### Study population

**Figure 1:**
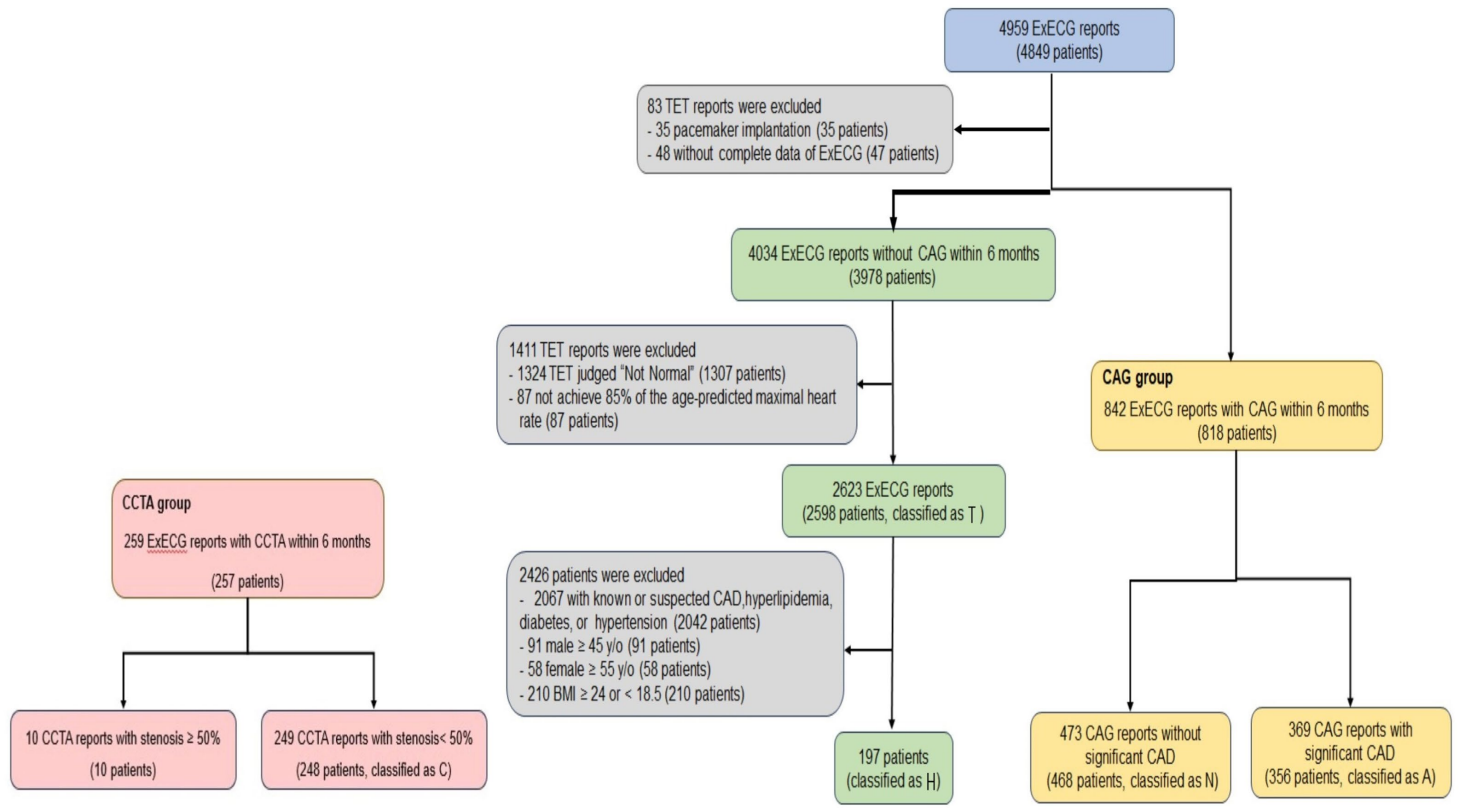
Flowchart of ExECG reports selection. This diagram outlined the systematic approach for selecting ExECG. TET = Treadmill exercise stress electrocardiographic test.

We enrolled 4,959 ExECG reports of 4,849 patients who underwent symptom-limited ExECG saved in XML format using the GE CASE 6.73 Stress Test system from January 2017 to January 2022 (Fletcher et al., 2013). We excluded patients with incomplete ExECG data or pacemaker implantation. The CAG group was defined as patients who underwent ExECG and subsequent CAG within 6 months, which was further divided into two subgroups with (A) and without (N) significant CAD. ExECG reports showing peak heart rates >85% of the maximum predicted rate and interpreted as normal by cardiologists, without subsequent CAG within 6 months, were categorized as subgroup T. In this group, patients with known or suspected CAD, hypertension, hyperlipidemia, diabetes, or clinical risk factors of CAD [male ≥45 years old, female ≥55 years old, or body mass index ≥24 or < 18.5 kg/m^2^] were further excluded, resulting in a subgroup at low risk of CAD (H). We selected patients who were evaluated through both ExECG and CCTA within 6 months at the Health Screening Center and had <50% coronary artery stenosis and classified them into subgroup C (Figure 1). Due to the variable positive predictive value of CCTA, ranging from 64 to 91%, patients identified by CCTA as having >50% coronary artery stenosis were excluded from the study (Arbab-Zadeh and Hoe, 2011). We included patients with a wide range of CAD severity to ensure the generalizability of our model.

We conducted model training using combinations of the aforementioned subgroups, resulting in five groups, including the CAG group (subgroups N and A); group II (subgroups N, A, and T); group III (subgroups N, A, and H); group IV (subgroups N, A, and C); and group V (subgroups N, A, H, and C). Some patients underwent more than one examination. We used any complete examination reports available, which resulted in a discrepancy between the number of examinations and patients.

Patients who underwent ExECG and CAG within 6 months at our institute and Asia University Hospital after February 2022 were used for external validation, respectively. The Institutional Review Board (IRB) of China Medical University Hospital approved this retrospective, single-center study (IRB number CMUH110-REC3-019).

### Coronary angiography

Coronary angiography was performed using standard techniques and used as the golden standard to determine the presence and severity of CAD. Significant CAD was defined as ≥50% stenosis of the left main stem and/or ≥ 70% stenosis in any major coronary artery (Nallamothu et al., 2013; Bhatt, 2015).

### ExECG data retrieval

**Figure 2:**
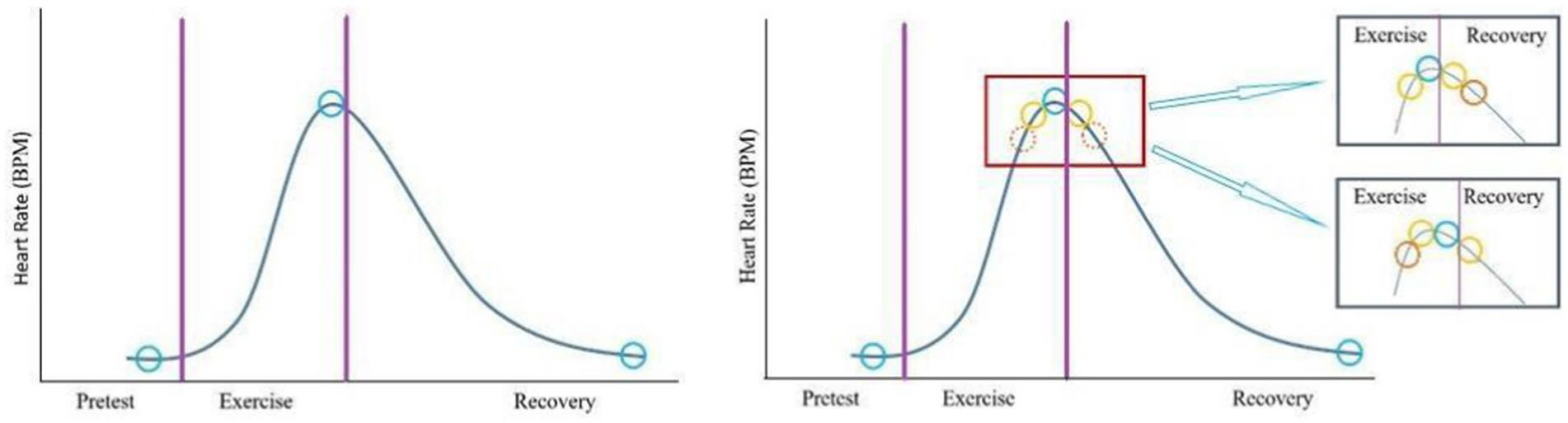
Selection of electrocardiographic slices. In model 1 (left panel), one slice (blue circle) of the signal in each stage was selected for training. In model 2 (right panel), three extra slices (yellow and solid orange circles) close to the peak heart rates during exercise and recovery phases were added.

The ExECG equipment generates a 10-s ECG signal at a frequency of 500 Hz in 12 ECG leads. ECGs in the pretest, exercise (peak heart rate), and recovery phases were retrieved for model 1 training (Figure 2, left panel). To investigate if the number of ECG signal slices for model input is proportional to the model’s capability, we added three extra slices close to the peak heart rate during the exercise and recovery phases for model 2 training (Figure 2, right panel).

The ExECG reports included supplementary physiological data alongside the ECG signals, which have been shown to enhance the accuracy of ExECG assessments (Lehtinen, 1999; Christman et al., 2014; Ahmed et al., 2015; Marzlin and Webner, 2019; Schultz et al., 2017; Ghaffari et al., 2017; Mieres et al., 2014; Snader et al., 1997). To improve the model’s effectiveness in detecting CAD, we integrated a carefully selected set of these physiological metrics as metadata, consisting of 14 primary features and two derived features. The primary features included sex, age, BMI, resting and peak heart rates, maximum predicted heart rate, resting and peak systolic and diastolic blood pressures, maximum rate-pressure product, maximum workload, maximum ST depression, and the ST/heart rate index. Additionally, we derived chronotropic incompetence and percent predicted metabolic equivalents to further support the model’s predictive capability (Supplementary material).

### ECG signals and metadata preprocessing

**Figure 3:**
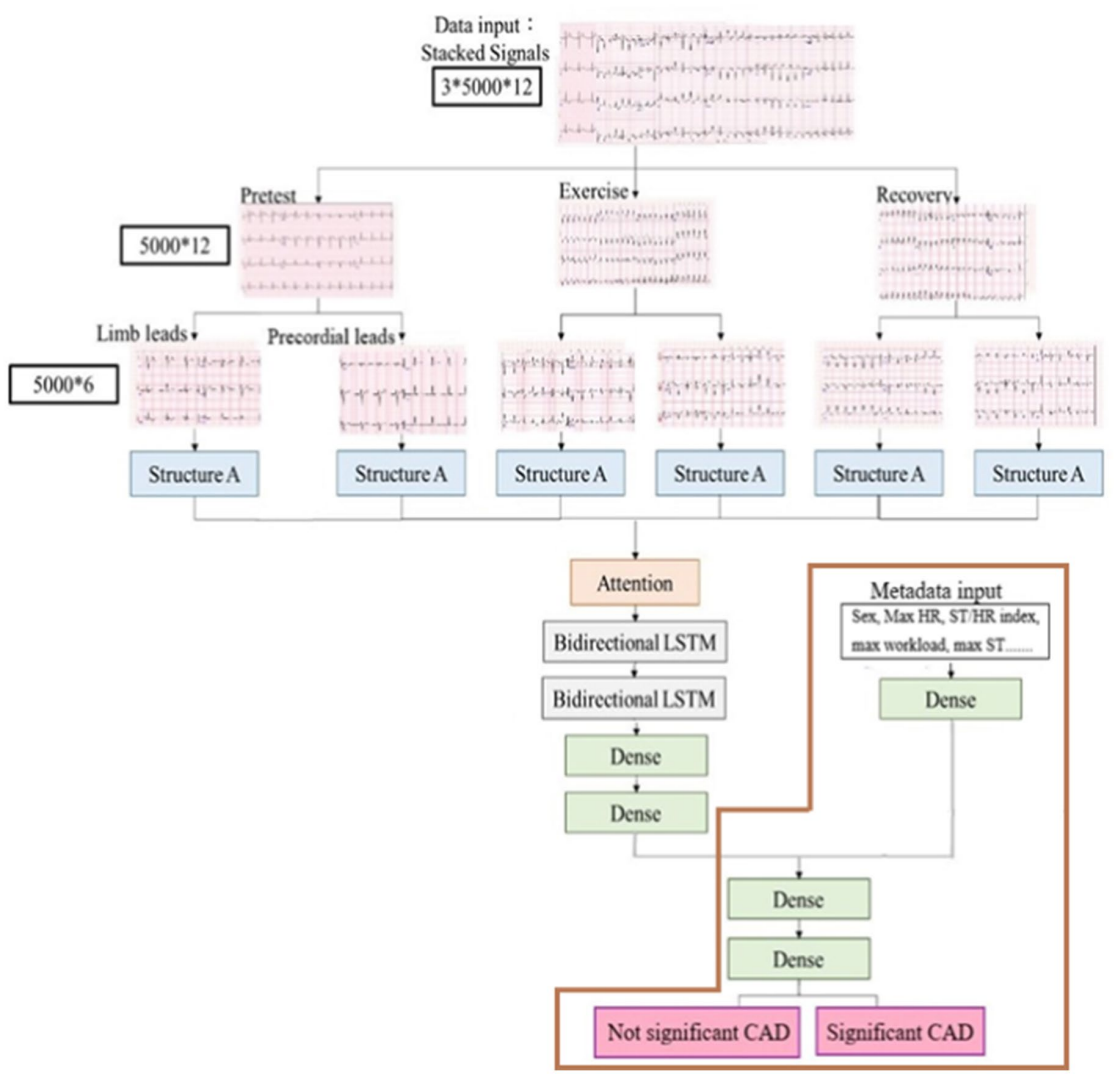
Process of ExECG analysis. It comprised data acquisition, preprocessing, feature extraction, and machine learning.

Our model processed input data as stacked ECG signals, annotated as 5,000 × 12 in three phases (pretest, exercise, and recovery), indicating that each lead had 5,000 data points. Subsequently, the ECG signals were separated into limb (I–III, aVR, aVL, and aVF) and precordial leads (V1–V6). Each lead, composed of one-dimensional data, was directed into an analytical module, structure A, for subsequent processing (Figure 3) (Yildirim et al., 2018; Kiranyaz et al., 2015).

Numerical and categorical variables of metadata were preprocessed using MinMaxScaler and one-hot encoder techniques. We excluded the ExECG if sex was absent. In our study, the missing data rate was minimal (0–0.5%), and the data followed a normal or near-normal distribution. For other variables with incomplete entries, we used the mean of the data, ensuring the continuity and integrity of the dataset for analysis. Outliers were individually reviewed to ensure accuracy. If it was not feasible to confirm whether an outlier was accurate, it was handled using the same approach as missing data.

### Model design

**Figure 4:**
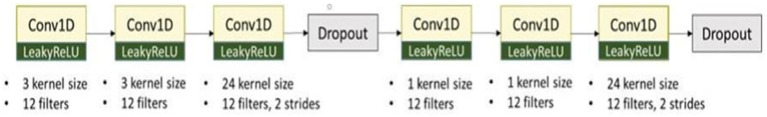
The architecture of structure A. It extracted the characteristics of ECG signals from limb and precordial leads in each phase and consisted of six layers of one-dimensional CNN layers.

For our model development, we designed a deep learning framework that integrated the CNN with LSTM network, as previously described (Chen et al., 2022) (Figure 3). Structure A in the architecture extracted the characteristics of ECG signals from the limb and precordial leads in each phase, and it consisted of six layers of one- dimensional CNN layers (Figure 4). We added a dropout layer after every three CNN layers, randomly discarding 20% of the information, to prevent overfitting. The Leaky Rectified Linear Unit (LeakyReLU) activation function was also used for each layer in structure A, which maintained the gradient flow during the training process, potentially leading to a better model performance. The output results from structure A were combined and input into the attention layer (Yang et al., 2016) to determine important weight vectors, followed by two bidirectional LSTM layers for sequence analysis. Subsequently, the output data were entered into two dense layers to classify the processed characteristics of ECG signals (Figure 3).

Additional physiological features (metadata) were also inputted into one dense layer. Subsequently, these outputs were integrated with the characteristics of ECG signals to serve as inputs of the judgment module, comprising of two dense layers and using sigmoid functions, culminating in the final determination of whether the patient had significant CAD (Figure 3). We further evaluated our model using only metadata, excluding ECG signals, as illustrated by the brown box in Figure 3.

### Model training

For each patient group, eligible ExECG reports were randomly divided into training, validation, and testing subsets in a 64:16:20 ratio. To maximize dataset utility, we employed K-fold cross-validation, a method particularly advantageous when data resources are limited. This approach splits the combined training and validation data (accounting for 80% of each group) into K equal folds. The model is trained on K − 1 folds, with the remaining fold used for validation, cycling through all folds. We set K to 5, implementing a 5-fold cross-validation process (Supplementary Figure 1).

### Performance evaluation

The performance metrics of the model were systematically assessed through various subgroup permutations. Our evaluative methodology included accuracy, AUC, sensitivity, specificity, positive and negative predictive values (PPV and NPV, respectively), and F1 score.

### Statistical analysis

Data were presented as mean ± standard deviation and percentages for continuous and categorical variables, which were compared using chi-square test and one-way analysis of variance, respectively. A two-sided *p* < 0.05 was considered statistically significant.

## Results

### Study population

Our study included 818 patients (842 ExECG reports) who underwent CAG (group CAG). Of these, 356 (369 CAG reports) and 468 patients (473 CAG reports) were identified with significant CAD (subgroup A) and not significant CAD (N), respectively. Moreover, 2,598 patients (2,623 ExECG reports) whose ExECG were interpreted as normal by cardiologists did not undergo subsequent CAG (T). We further excluded individuals with risk factors of CAD, leading to 197 subjects at low risk of CAD (H). Additionally, 248 patients (249 CCTA reports) whose CCTA showed <50% coronary artery stenosis were classified as subgroup C (Figure 1). Table 1 shows the clinical and demographic characteristics of both CAG and non-CAG groups. The mean age in subgroup A was 59.0 ± 9.8 years, which was older than the other subgroups. Additionally, subgroup A had a higher prevalence of male sex, hypertension, diabetes, and hyperlipidemia. We used 325 and 114 patients at our institute after February 2022 and Asia University Hospital for external validation, respectively.

**Table 1:** Clinical and demographic characteristics.

Characteristic	Non-CAG group			CAG group	
Patient, *n*	C = 248*	H = 197	T = 2598*	N = 468*^,#^	A = 356*^,#^
ExECG, *n*	249	197	2,623	473	369
CAG or CCTA, *n*	249	0	0	473	369
Age (yrs)	52.6 ± 11.6	34.4 ± 10.3	48.2 ± 13.8	54.7 ± 11.6	59.0 ± 9.8
Male, *n* (%)	128 (52%)	80 (41%)	1,483 (57%)	299 (64%)	315 (88%)
Hypertension, *n* (%)	138 (56%)	0 (0%)	1,288 (50%)	328 (70%)	309 (87%)
Diabetes, *n* (%)	24 (10%)	0 (0%)	236 (9%)	75 (16%)	110 (31%)
Hyperlipidemia, *n* (%)	93 (38%)	0 (0%)	600 (23%)	189 (40%)	197 (55%)
BMI (kg/m^2^)	24.5 ± 3.3	21.4 ± 1.4	24.9 ± 4.1	25.6 ± 4.0	26.2 ± 3.2

*One patient in subgroup C, 25 in T, 5 in N, and 13 in A underwent examinations twice. ^#^Six patients were included in both A and N subgroups according to CAG reports.

### Quantity of ECG slice and features

**Figure 5:**
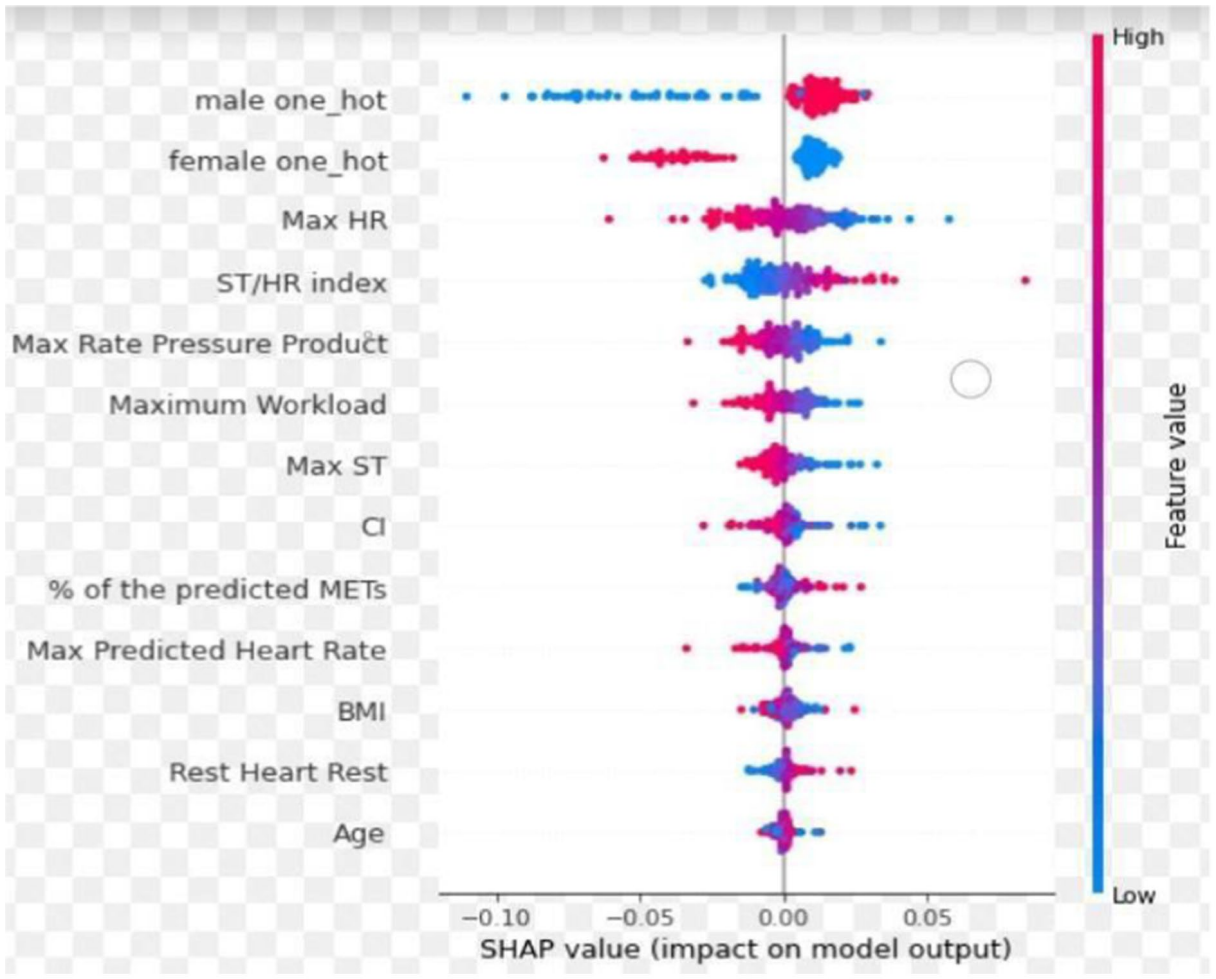
The SHapley additive exPlanations summary plot. The red and blue colors represent the feature values, with red indicating high feature values and blue corresponding to low feature values. Positive SHAP values indicate that the feature increases the likelihood of predicting significant CAD, while negative SHAP values suggest a decreased likelihood of significant CAD. Sex, maximum HR and ST/HR index were the most significant predictors in our model.

In the CAG group (A + N), optimal performance was achieved by integrating three ECG slices (pretest, peak heart rate, and recovery phases) and 12 features without blood pressure data, resulting in AUC, sensitivity, and specificity of 0.74, 0.86, and 0.47, respectively (Table 2). More slices and/ or features did not improve model performance. The SHapley Additive exPlanations summary plot (Figure 5) showed that sex, maximum HR, and ST/HR index were the most significant predictors in our model. Therefore, we used the integration of three ECG slices and 12 features in the next stage.

**Table 2:** Model performance across varied quantities of inputted ECGs and features.

Inputted number		Model performance				
ECGs	Features	ACC	AUC	F1	SEN	SPE
N = 6	0	0.62	0.66	0.58	0.58	0.64
	10	0.64	0.66	0.59	0.57	0.69
	12	0.64	0.69	0.68	0.83	0.47
	14	0.65	0.70	0.68	0.82	0.50
	16	0.68	0.72	0.68	0.75	0.61
N = 3	0	0.65	0.68	0.66	0.72	0.59
	10	0.60	0.68	0.65	0.79	0.44
	12	0.65	**0.74**	0.70	**0.86**	**0.47**
	14	0.66	0.72	0.64	0.65	0.68
	16	0.64	0.71	0.68	0.82	0.48

ACC = accuracy; AUC = area under the curve; F1 = F1 score; SEN = sensitivity; SPE = specificity. Bold value indicated optimal performance.

### Performance across five training groups

Subsequently, the model was trained using various group combinations. The outcomes across five training groups were not significantly different if the test set only included the CAG group (A + N) (Table 3), with AUC, sensitivity, and specificity of 0.74–0.78, 0.82–0.89, and 0.47–0.51, respectively. When T subgroup was integrated into the training and test sets (group II), accuracy significantly improved, with AUC, NPV, and specificity of 0.75, 0.88, 0.97, and 0.75, respectively. However, this enhancement was achieved with detriment to the F1, and PPV. When low-risk patient data (H) were included for both training and testing (group III), accuracy significantly improved, with AUC, NPV, and specificity of 0.71, 0.83, 0.90, and 0.60, respectively. However, the F1 score, PPV, and sensitivity had minimal variation. The demographic, CAG, and ExECG features did not differ significantly between training, validation, and testing sets in group III (Supplementary Table 1). The performance was not significantly different when the model was trained and tested on groups III, IV, or V (Table 3). We also compared our models with the conventional ExECG algorithm, which primarily focuses on exercise-induced ST-segment changes as assessed by board-certified cardiologists with varying levels of clinical experience (Mieres et al., 2014). Compared with the conventional ExECG algorithm, the performance of our AI model showed improvements across all measured variables and delivered predictive outcomes within 1 min after completing ExECG.

**Table 3:** Performance comparison of the model integrating ECG signals and metadata across subgroup combinations.

Training set	Test set	ACC	AUC	F1	PPV	NPV	SEN	SPE
Gr CAG, N: A	CAG, N: A	0.65	0.74	0.70	0.59	0.79	0.86	0.47
	N: A*	0.63	0.74	0.69	0.58	0.73	0.84	0.42
	N: A^#^	0.55	0.70	0.62	0.49	0.73	0.84	0.34
Gr II, (T + N): A	CAG, N: A	0.66	0.76	0.69	0.60	0.77	0.82	0.51
	N: A*	0.59	0.73	0.66	0.56	0.69	0.83	0.37
	N: A^#^	0.61	0.68	0.66	0.53	0.82	0.88	0.42
	II, (T + N): A	0.75	0.88	0.43	0.29	0.97	0.82	0.75
Gr III, (H + N): A	CAG, N: A	0.66	0.78	0.71	0.59	0.82	0.89	0.47
	N: A*	0.61	0.75	0.69	0.57	0.77	0.89	0.35
	N: A^#^	0.55	0.63	0.63	0.49	0.77	0.88	0.31
	III, (H + N): A	0.71	0.83	0.69	0.57	0.90	0.89	0.60
Gr IV, (C + N): A	CAG, N: A	0.67	0.78	0.71	0.60	0.83	0.89	0.48
	N: A*	0.62	0.75	0.69	0.57	0.78	0.89	0.35
	N: A^#^	0.56	0.63	0.63	0.49	0.78	0.88	0.32
	IV, (C + N): A	0.65	0.79	0.65	0.51	0.89	0.89	0.52
Gr V, (C + H + N): A	CAG, N: A	0.66	0.78	0.71	0.59	0.82	0.89	0.47
	N: A*	0.62	0.75	0.69	0.57	0.78	0.89	0.35
	N: A^#^	0.56	0.63	0.63	0.49	0.78	0.88	0.32
	V, (C + H + N): A	0.68	0.82	0.63	0.49	0.92	0.89	0.59
Conventional	CAG, N: A	0.46	-	0.55	0.45	0.49	0.71	0.24

* External validation from data of our institute after Feb 2022. # External validation from data of Asia university hospital. ACC = accuracy; AUC = area under the ROC curve; F1 = F1 score; PPV = positive predictive value; NPV = negative predictive value; SEN = sensitivity; SPE = specificity.

The performance of the model using the combination of ECG signals and metadata was comparable to that using metadata alone (the brown box in Figure 3), as shown in Table 4. However, the model integrating ECG signals and metadata demonstrated higher sensitivity compared to the model relying solely on metadata.

**Table 4:** Comparison of model performance using ECG signals and metadata versus metadata alone.

Patients	Model	ACC	AUC	F1	PPV	NPV	SEN	SPE
Gr CAG, N: A	ECG+ metadata	0.65	0.74	0.70	0.59	0.79	0.86	0.47
	Only metadata	0.66	0.72	0.67	0.61	0.72	0.73	0.59

#### External validation

The comparative analysis of performance across five training groups showed minimal discrepancy in patients at our institute after February 2022 and Asia University Hospital (Table 3), indicating that our models can potentially be applied in diverse settings.

#### Bootstrap validation

**Figure 6:**
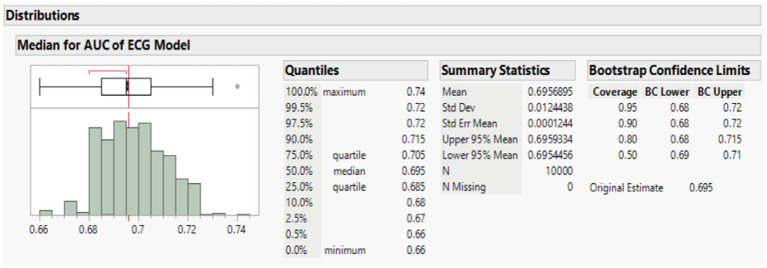
The bootstrap validation showed a stable mean AUC with narrow confidence intervals.

We implemented bootstrapping to assess low AUC probability of Table 2 and simulated the sample size improvement (*n* = 10,000) with a flexible bootstrap distribution. The results showed that the AUC distribution of our model was stable, with minimal variability and a narrow confidence interval. The consistency between bootstrap estimates and the original value underscored the reliability of this method for performance validation using SAS JMP Academic Suite Version 17.2 (JMP Inc., NC, United States) (Figure 6).

## Discussion

Our study provides an efficient and accurate tool to identify patients with significant CAD by the AI-enhanced ExECG algorithm, which achieved an AUC of 0.83, a sensitivity of 0.89, and a specificity of 0.60, within 1 min. The most important feature predictors for our model performance were sex, maximum heart rate, and ST/HR index.

The conventional ExECG algorithm mainly depends on ST segment changes (Gibbons et al., 2002). A meta-analysis of 147 studies involving 24,047 patients reported mean sensitivity and specificity of 68 and 77%, respectively, but with considerable variability, ranging from 23–100% for sensitivity and 17–100% for specificity. The variation in diagnostic accuracy could be attributed to significant disparities in the demographic and clinical profiles of the studied cohorts, divergent criteria for defining the presence and severity of CAD, and differences in the selection of diagnostic variables (Fletcher et al., 2013; Detrano et al., 1989). Compared with the conventional ExECG algorithm, our model, incorporating ECG signal along with 12 features, showed superior performance.

In 2009, Babaoglu et al. initially explored the use of AI algorithms to detect and localize CAD through ExECG (Babaoglu et al., 2009). Their methodology incorporated 27 distinct features as inputs into their model. Subsequently, they refined their approach by reducing the feature set to 18 and applied the support vector machine method for further studies (Babaoğlu et al., 2010). Various models have been developed with the rapid evolution of machine learning technologies. Lee et al. introduced the random forest algorithm to enhance ExECG diagnostic capabilities, utilizing a dataset comprising 30 specific features, with the option to incorporate clinical data (Lee et al., 2022). Following this advancement, Yilmaz et al. implemented the eXtreme gradient boosting algorithm, capitalizing on ECG characteristics and signals presented in JPEG format for their analysis (Yilmaz et al., 2023). Compared with a previous study, our training model using five different patient groups showed not inferior performance, supported by AUC metrics, when assessed in the CAG group (Table 5). The AUC values reported in these ExECG-CAG studies were not as high as those observed in other AI implementations for ECG analyses, such as those for arrhythmia and systolic dysfunction (Attia et al., 2019; Adedinsewo et al., 2020). The selection bias inherent in ExECG-CAG studies might account for this discrepancy. Specifically, patients selected for CAG typically showed a higher probability of having obstructive CAD, a selection criterion that usually excludes healthy individuals. Consequently, this predisposition influenced the severity spectrum used during model training, culminating in acceptable but not outstanding AUC values.

**Table 5:** The comparison with the existing literature.

Study	Cohort and definition of CAD	Input	Model	Output	ACC	AUC	SEN	SPE
Babaoglu et al. (2009)	**Training and test cohorts** 330 patients with ExECG and CAG within one month **Definition of CAD** Narrowing ≥50% in LM, or narrowing ≥70% in the other major coronary arteries	27 features from the ExECG data	ANN	LM	0.91	-	0.25	0.97
				LAD	0.73	-	0.75	0.70
				LCX	0.65	-	0.50	0.75
				RCA	0.69	-	0.50	0.80
Babaoğlu et al. (2010)	**Training and test cohorts** 480 patients with ExECG and CAG within a month **Definition of CAD** Narrowing ≥50% in LM, or narrowing ≥70% in the other major coronary arteries	18 features from the ExECG data using the PCA method	SVM	CAD	0.79	-	-	-
Lee et al. (2022)	**Training and test cohorts** 2,325 patients with ExECG and CAG within a year **Definition of CAD** narrowing ≥70% in LAD, LCX, RCA, or their main branches, or narrowing ≥50% in LM	30 features from the ExECG data	RF	CAD	0.57	0.73	0.85	0.43
		30 features from the ExECG data and 7 clinical features		CAD	0.59	0.74	0.85	0.45
Yilmaz et al. (2023)	**Training and test cohorts** 294 patients with ExECG and CAG in the same month. **Definition of CAD** narrowing ≥70% in LAD, LCX, RCA, or LM	23 extracted features from ExECG signal in JPEG format	XGBoost	CAD	0.81	0.78	0.67	0.85
Our study	**Training and test cohorts** 842 patients with ExECG and CAG within 6 months **Definition of CAD** Narrowing ≥50% in LM, or narrowing ≥70% in the other major coronary arteries	ExECG signals in XML format and 12 features	CRNN	CAD	0.65	0.74	0.86	0.47

CAD = coronary artery disease; ACC = accuracy; AUC = area under the ROC curve; SEN = sensitivity; SPE = specificity; ExECG = exercise electrocardiography; CAG = coronary angiography; LAD = left anterior descending artery; LCX = left circumflex artery; RCA = right coronary artery; LM = left main segment; XGBoost = eXtreme Gradient Boosting; RF = Random Forest; SVM = Support Vector Machine; ANN = Artificial Neural Network; CRNN = Convolutional Recurrent Neural Networks.

Healthy individuals are commonly included into the training sets to enhance the generalization capability of AI models, facilitating its broader applicability across patients at various risks (Huang et al., 2022). Thus, we included individuals with normal ExECG interpreted by cardiologists and those exhibiting insignificant coronary artery stenosis determined by CCTA into the training sets (groups II–V) (Table 3). The performance metrics for each group (groups II–V) significantly improved, with AUC, sensitivity, and specificity of 0.79–0.88, 0.82–0.89, and 0.52–0.75, respectively. Although accuracy and AUC were the highest when the model underwent both training and testing on group II, PPV and F1 score significantly decreased, with increased specificity. This observation suggests a propensity for the model, when trained with data from group II, to exhibit a bias towards classifying subjects as normal. This alteration may be attributed to data imbalance, considering that the sample size of subgroup T disproportionately exceeded that of subgroup A (Huang et al., 2023). Furthermore, the possibility of silent ischemia in subgroup T could not be entirely ruled out, potentially contributing to a reduction in both PPV and F1 score. Conversely, the outcomes of the model trained and tested on group III including patients at low risk (H) also showed excellent discrimination, supported by an AUC, sensitivity, and specificity of 0.83, 0.89, and 0.60, respectively, which was achieved without compromising the PPV and F1 score. Considering that coronary artery disease is treatable yet often presents acutely and can lead to severe complications, we prioritized designing an algorithm with greater sensitivity and accuracy to assist in the early identification of potential cases. The performance of the model when using group IV or V did not surpass that noted in group III. This observation may be due to the patients undergoing CCTA (included within groups IV or V) are generally older and possess higher cardiovascular disease risk factors, aligning more closely with the characteristics of subgroup A instead of subgroup H (Table 1). These demographic and clinical characteristics may potentially lead to model misinterpretation. Similarly, although the performance of the model using only metadata was comparable to that of the model integrating ECG signals and metadata (Table 4), we prioritized higher sensitivity, which led to our selection of the model combining ECG signals and metadata.

The ECG images presented in PDF or JPEG formats in previous studies underwent processing by the equipment and limited the display of each lead to 2.5 s. In contrast, we used original ECG signals directly generated by the equipment, extending the duration for each lead to 10 s, which enhanced the model’s access to comprehensive ECG data. By combining CNNs and LSTMs into a CRNN architecture, our model provides the benefit of both spatial feature extraction and temporal sequence modeling, allowing our model to understand the complex structure of ECG data, recognizing the immediate patterns in the signals and how these patterns change over time (Verma and Agarwal, 2018; Zhang et al., 2020; Zihlmann et al., 2017). In our investigation, the analysis of three ECG signal slices with 12 specific features during the pretest, peak heart rate, and recovery phase yielded optimal performance metrics. However, the additional ECG slices or features did not enhance predictive outcomes. The principal predictive variables were sex, maximum heart rate, and ST/HR index (Figure 5), offering valuable insights into the weighting of features to identify significant CAD (Lehtinen, 1999; Christman et al., 2014; Ahmed et al., 2015; Marzlin and Webner, 2019; Schultz et al., 2017; Ghaffari et al., 2017; Mieres et al., 2014; Snader et al., 1997). Moreover, our model can generate results within 1 min after completing ExECG. Future research should aim to enhance specificity by integrating clinical and imaging data, optimizing the AI algorithm, applying differential weighting during training, incorporating additional physiological features from the ExECG report, exploring the impact and significance of metadata, and expanding training datasets to include larger and more diverse populations (Benkarim et al., 2022; McKinney et al., 2020; Sato et al., 2022; Marwick et al., 1995; Gencbay et al., 1999; Siegler et al., 2011).

### Limitations

Our study has limitations. First, the patient cohort was recruited from a single institution; notwithstanding, external validation was conducted at Asia University Hospital, revealing minimal variance in performance outcomes across the two facilities. Second, angiographic analyses were conducted by interventional cardiologists engaged in routine clinical practice rather than by a dedicated core laboratory, which may introduce a degree of subjective bias in interpretation. Despite these, significant stenosis was accurately identified consistently. Third, not all participants underwent angiography or CCTA; however, the likelihood of erroneously classifying patients with significant CAD as normal was reduced by preferentially selecting individuals at low risk for CAD. Additionally, our study focused on epicardial stenosis and did not assess coronary microvascular dysfunction. In some patients with diabetes or hypertension, coronary artery disease may arise from microvascular dysfunction rather than macrovascular stenosis (Vrints et al., 2024; O'Neal et al., 2017; Angeja et al., 2002; Okin et al., 2004). Using CAG-detected epicardial stenosis as the gold standard for evaluating functional tests like ExECG may not fully reflect the underlying pathophysiology and could negatively affect AI performance. Finally, low PPV and specificity might increase the incidence of unnecessary CAG. This limitation is primarily attributable to the selection bias inherent in ExECG-CAG studies. Moreover, our primary objective is to assist physicians in efficiently screening patients following ExECG, rather than acting as the sole determinant for advanced invasive testing. Therefore, when AI-generated findings raise clinical uncertainty, additional imaging modalities should be considered. Further research aimed at improving specificity is warranted.

## Conclusion

Our AI-based algorithm has shown promise in identifying patients with significant CAD using ExECG data. Integrating a multimodal approach that combines ECG signals with additional features enhances both predictive performance and efficiency. Further large-scale studies and algorithm refinements are needed to improve specificity and validate clinical utility across diverse patient populations.

### Summary

This study aimed to develop an artificial intelligence (AI)-based method to enhance the efficiency and accuracy of exercise stress electrocardiography (ExECG) in detecting significant coronary artery disease (CAD). We retrospectively analyzed 818 patients who underwent both ExECG and coronary angiography (CAG) within 6 months. We used a Convolutional Recurrent Neural Network algorithm, which integrated electrocardiographic (ECG) signals and ExECG report features to predict significant CAD. The algorithm achieved an area under the curve (AUC) of 0.74, sensitivity of 0.86, and specificity of 0.47. With the inclusion of 197 low-risk patients, AUC, sensitivity, and specificity improved to 0.83, 0.89, and 0.60, respectively. Optimal performance was achieved with three ECG signal slices and 12 features, including sex, maximum heart rate, and ST/HR index as principal predictive variables. The AI model generated results within 1 min after completing ExECG, suggesting its potential to identify significant CAD efficiently and accurately in both symptomatic and asymptomatic patients, thereby enhancing clinical screening.

## Data Availability

The raw data supporting the conclusions of this article will be made available by the authors, without undue reservation.
