# Supplementary material for: Deep learning analysis of exercise stress electrocardiography for identification of significant coronary artery disease
Source: Front Artif Intell. 2025 Mar 17;8:1496109. doi: 10.3389/frai.2025.1496109 (PMC11955648; doi:10.3389/frai.2025.1496109)
Supplement: Supplementary file 1 [file Data_Sheet_1.docx]

- Chronotropic Incompetence

= [ (Peak Heart Rate- Rest Heart Rate)/ (220-Age- Rest Heart Rate)]×100%

- Percent predicted metabolic equivalents

= [ (maximum METs)/(predicted METs)]×100%

with Predicted METs = [18.0–(0.15×Age) ]for male;

= [14.7–(0.13×Age)] for female

**Supplementary Figure 1**


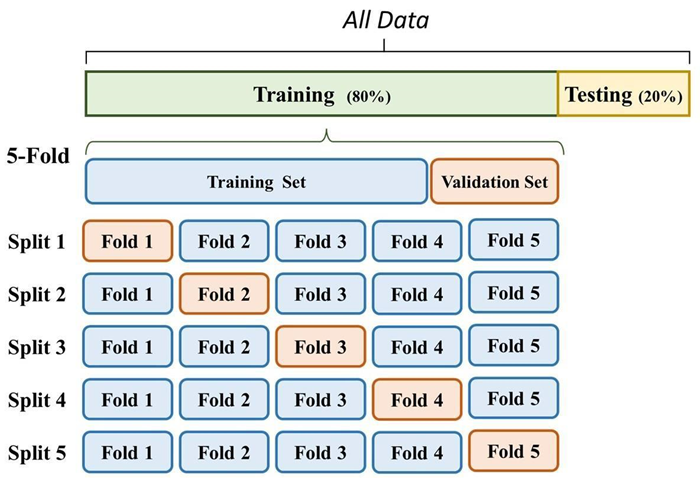


**Figure** The K-fold cross-validation. In this study, K was established at 5, facilitating a 5-fold cross-validation process. In Split 1, Fold 1 was used as the validation set, while Folds 2 through 5 were used as the training set.

Supp Table 1. Demographic characteristics of training, validation, and testing sets utilizing group III

| ExECG number | Total  (N= 1039) | Training Set (N=664) | Validation Set (N=165) | Testing Set  (N=210) | P value |
| --- | --- | --- | --- | --- | --- |
| Age (yrs) | 52.3±13.9 | 52.0±13.7 | 54.2±13.8 | 51.9±14.7 | 0.16 |
| Male, n (%) | 711(68%) | 465 (70%) | 111 (67%) | 135 (64%) | 0.28 |
| BMI (kg/m^2^) | 25.0±3.8 | 25.0±3.9 | 25.0±3.8 | 24.9±3.6 | 0.84 |
| Resting HR (beats/min) | 86±13 | 86±14 | 84±12 | 88±14 | 0.03 |
| Peak HR (beat/min) | 151±20 | 151±20 | 149±21 | 150±19 | 0.41 |
| Max Predicted HR (%) | 89.4±9.5 | 89.6±9.7 | 89.4± 10.4 | 88.9± 8.5 | 0.65 |
| Resting Sys BP (mmHg) | 131±19 | 130±19 | 133±18 | 130±19 | 0.36 |
| Resting Dia BP (mmHg) | 79±12 | 79±12 | 80±11 | 78±12 | 0.51 |
| Peak Sys BP (mmHg) | 180±25 | 180±25 | 179±25 | 182±25 | 0.51 |
| Peak Dia BP (mmHg) | 79±15 | 79±15 | 81±16 | 78±15 | 0.33 |
| Max Rate Pressure Product | 25126±5548 | 25185±5526 | 24490±5648 | 25434±5526 | 0.24 |
| Maximum Workload (Mets) | 9.1±2.4 | 9.1±2.4 | 9.1±2.8 | 9.1± 2.3 | 0.95 |
| Max ST depression (mm) | -1.8±1.1 | -1.8±1.1 | -1.7±1.1 | -1.7±1.1 | 0.75 |
| ST/HR index | 1.9±2.0 | 1.9±2.0 | 1.9± 2.2 | 1.8±1.8 | 0.98 |
| Chronotropic Incompetence (%) | 79.3±19.8 | 79.8±20.3 | 79.2±22.5 | 77.5±15.8 | 0.35 |
| Percent predicted metabolic equivalents | 1.0±0.2 | 1.0±0.2 | 1.0±0.3 | 1.0±0.2 | 0.37 |
| Significant CAD, n (%) | 670 (64%) | 428 (64%) | 111 (67%) | 131 (62%) | 0.62 |
| Bruce Protocol (%) | 97% | 97% | 96% | 96% | 0.58 |
| Mean BRUCE duration(m:s) | 07:31 | 07:31 | 07:24 | 07:34 | 0.78 |

HR= heart rate; Sys= systolic; Dia= diastolic; BP= blood pressure; CAD= coronary artery disease;

P value between training, validation and test cohorts
